# Use of CHAID Decision Trees to Formulate Pathways for the Early Detection of Metabolic Syndrome in Young Adults

**DOI:** 10.1155/2014/242717

**Published:** 2014-04-10

**Authors:** Brian Miller, Mark Fridline, Pei-Yang Liu, Deborah Marino

**Affiliations:** ^1^School of Nutrition and Dietetics, College of Health Professions, The University of Akron, Akron, OH 44325-6102, USA; ^2^Department of Statistics, College of Arts and Sciences, University of Akron, Akron, OH 44325-1913, USA

## Abstract

Metabolic syndrome (MetS) in young adults (age 20–39) is often undiagnosed. A simple screening tool using a surrogate measure might be invaluable in the early detection of MetS. *Methods*. A chi-squared automatic interaction detection (CHAID) decision tree analysis with waist circumference user-specified as the first level was used to detect MetS in young adults using data from the National Health and Nutrition Examination Survey (NHANES) 2009-2010 Cohort as a representative sample of the United States population (*n* = 745). *Results*. Twenty percent of the sample met the National Cholesterol Education Program Adult Treatment Panel III (NCEP) classification criteria for MetS. The user-specified CHAID model was compared to both CHAID model with no user-specified first level and logistic regression based model. This analysis identified waist circumference as a strong predictor in the MetS diagnosis. The accuracy of the final model with waist circumference user-specified as the first level was 92.3% with its ability to detect MetS at 71.8% which outperformed comparison models. *Conclusions*. Preliminary findings suggest that young adults at risk for MetS could be identified for further followup based on their waist circumference. Decision tree methods show promise for the development of a preliminary detection algorithm for MetS.

## 1. Introduction

Metabolic Syndrome (MetS) is a collection of cardiometabolic risk factors that includes excessive central adiposity, elevated triglycerides (TG) and fasting plasma glucose (FPG), decreased HDL-cholesterol (HDL), and hypertension [1]. When these risk factors are present in tandem, they increase the risk of heart attack, stroke, and cardiovascular morbidity and/or mortality affecting one in three adults in the United States (US) [2]. Additionally, there is a disproportionate increase in healthcare costs for adults presenting with MetS compared to those that do not [3, 4]. Prevalence and complications associated with MetS and other cardiometabolic diseases continue to be a major health concern in the United States.

The National Cholesterol Education Program Adult Treatment Panel III (NCEP) and International Diabetes Federation (IDF) clinical risk models are limited in their usefulness in that they only identify either the presence or absence of MetS [3, 4]. However much like obesity, there are varied clinical implications based on the severity of the risk factors used to define MetS. Furthermore, certain factors might be more significant than others in predicting the presence or absence of MetS. Waist circumference has been demonstrated to be a strong predictor of cardiometabolic risk [2, 5, 6] and can be easily and affordably obtained in a clinical screening. Creating an early detection model that stratifies the severity of cardiometabolic and anthropometric factors used in the MetS diagnosis based on proxy measures easily obtained in a clinical setting would be invaluable for clinicians aiming to provide improved patient-centered care [7].

Predictive models are useful and cost effective in identifying risk of developing cardiometabolic chronic diseases [8]. Decision tree methodologies show promise over traditional predictive modeling procedures based on their ease of interpretability by nonstatisticians. One of the outstanding advantages of decision tree analysis is that it can visualize the relationship pathways between the binary target variable and the related continuous and/or categorical predictor variables with a tree image [9]. Recently, Worachartcheewan et al. [10] used a classification and regression tree (CART) model to identify pathways for MetS detection in accordance with the NCEP criteria using a large Thai population of overweight men and women without regard to age or health status. In this model, TG was the strongest predictor of MetS. However, dyslipidemia is not commonly elevated in younger adulthood and is invasive and costly to measure [7].

Unfortunately, there is a lack of research focusing on the adult population ages of 20–39 years where preventative or early corrective measures can be utilized. Rather, the majority of research has focused on the adult population greater than age 40 [11, 12]. Currently no preventative methodologies exist for the early detection of MetS. Therefore, attention is warranted to the derivation of premetabolic syndrome criteria that identifies at-risk subjects who can utilize preventive intervention well before qualifying as moderate to high risk on current predictive models [13].

The purpose of this pilot study is to investigate the utility of the chi-squared automatic interaction detection (CHAID) algorithm to identify and develop pathways for the early detection of MetS. The central hypothesis states that the decision tree pathways derived from CHAID algorithms using data from National Health and Nutrition Examination Survey (NHANES) 2009-2010 will detect the presence of MetS in adults of 20–39 years of age. These pathways are meant to serve as pilots for the future development of an easily interpreted, clinically relevant, cost-effective screening tool to detect cardiometabolic chronic disease [14].

## 2. Materials and Methods

### 2.1. Participants

The current study is based on publicly available data from the National Health and Nutrition Examination Survey (NHANES) 2009-2010 cohort [15]. The full data set includes 10,537 subjects designed to represent the population of the United States across age, sex, and ethnicity. Subjects with missing MetS criteria were excluded from the present study due to the inability in making a complete classification of MetS (subjects lost *n* = 7589). Subjects not meeting the inclusion criteria of an age between 20 and 39 years were excluded as were those with a body mass index (BMI) less than 20 kg/m² (subjects lost *n* = 2203; *n* = 522 for age <20 years, *n* = 1622 for age >39 years, and *n* = 59 for BMI <20 kg/m²). The final sample retained meeting the inclusion criteria included 745 subjects.

Demographic information included age, sex, and dichotomous ethnicity represented as ethnic or nonethnic. Anthropometric information included weight (kg), height (cm), BMI (kg/m²), and waist circumference (cm). Laboratory measures included HDL (mg/dl), TG (mg/dl), fasting plasma glucose (FPG, mg/dl), and blood pressure expressed as systolic and diastolic pressures (mmHg).

The criteria for MetS followed the NCEP guidelines defined as presenting with three or more of the following factors: waist circumference > 88 cm for women or >102 cm for men, blood pressure ≥ 135/≥85 mmHg, TG ≥ 150 mg/dl, HDL < 50 mg/dl for women or < 40 mg/dl for men, or FPG ≥100 mg/dl [16]. Sample characteristics are illustrated in Table 1 and are expressed as mean ± standard deviation. Of the 745 subjects between the ages of 20–39 years, 20% (*n* = 149) presented with the NCEP criteria for MetS. Approval for this analysis was provided by the University of Akron Institutional Review Board.

### 2.2. Statistical Analysis

The data was arranged in a column-wise format with each subject given a sequence identifier. Data management was performed using data set merging and data subset functions with statistical analysis performed using IBM SPSS version 19. A CHAID algorithm analysis was used to develop the decision tree models. CHAID decision trees are nonparametric procedures that make no assumptions of the underlying data. This algorithm determines how continuous and/or categorical independent variables best combine to predict a binary outcome based on “if-then” logic by portioning each independent variable into mutually exclusive subsets based on homogeneity of the data. For this study, the response variable is the presence or absence of MetS. According to Kass (1980), the CHAID algorithm operates using a series of merging, splitting, and stopping steps based on user-specified criteria as follows [17].

The merging step operates using each predictor variable where CHAID merges nonsignificant categories using the following algorithm.

1. Perform cross-tabulation of the predictor variable with the binary target variable.
2. If the predictor variable has only 2 categories, go to step 6.
3. *χ*²-test for independence is performed for each pair of categories of the predictor variable in relation to the binary target variable using the *χ*² distribution (df = 1) with significance (*α*_merge_) set at 0.05. For nonsignificant outcomes, those paired categories are merged.
4. For nonsignificant tests identified by *α*_merge_ > 0.05, those paired categories are merged into a single category. For tests reaching significance identified by *α*_merge_ ≤ 0.05, the pairs are not merged.
5. If any category has less than the user-specified minimum segment size, that pair is merged with the most similar other category.
6. The adjusted *P* value for the merged categories using a Bonferroni adjustment is utilized to control for Type I error rate.

The splitting step occurs following the determination of all the possible merges for each predictor variable. This step selects which predictor is to be used to “best” split the node using the following algorithm.

1. *χ*²-test for independence using an adjusted *P* value for each predictor.
2. The predictor with the smallest adjusted *P* value (i.e., most statistically significant) is split if the *P* value less than the user-specified significance split level (*α*_split_) is set at 0.05; otherwise the node is not split and is then considered a terminal node.

The stopping step utilizes the following user-specified stopping rules to check if the tree growing process should stop.

1. If the current tree reached the maximum tree depth level, the tree process stops.
2. If the size of a node is less than the user-specified minimum node size, the node will not be split.
3. If the split of a node results in a child node whose node size is less than the user-specified minimum child node size value, the node will not be split. The parent node is the level where the data set divides into child nodes that can themselves become either parent nodes or end in a terminal or decision node.
4. The CHAID algorithm will continue until all the stopping rules are met.

The CHAID analysis was run in duplicate with parent nodes defined at 20 subjects, child node defined at 5 subjects, and significance set at (*α*_merge_, *α*_split_, and *P* value) ≤0.05.

For the first run, the first level or first division was user-specified as waist circumference due to the measurement of this parameter having the lowest cost in MetS screening [18, 19]. The second run was utilized as a comparison to the first model with no first division user-specified. This allowed the algorithm to determine the parameter of the first split. CHAID accuracy and detection was expressed as percentages.

Logistic regression with testing for multicollinearity was performed on the five factors used to define MetS as a parametric comparison to the CHAID models. Results were expressed as overall accuracy of the logistic regression model and detection of MetS, both expressed as percentages with significance of the overall model set at *P* ≤ 0.05.

## 3. Results

### 3.1. CHAID: Waist Circumference User-Specified

**Figure 1:**
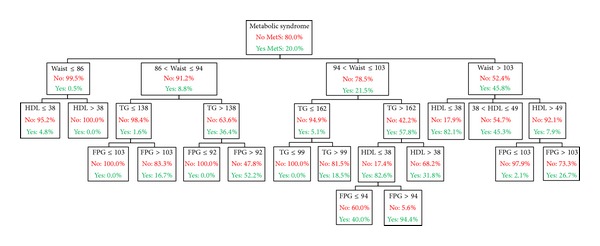
MetS: metabolic syndrome, TG: triglyceride (mg/dl), HDL: high-density lipoprotein cholesterol (mg/dl), Waist: waist circumference (cm), and FPG: fasting plasma glucose (mg/dl).

The decision tree algorithm partitioned the data into statistically significant subgroups that were mutually exclusive and exhaustive [17]. The tree analysis in Figure 1 shows the 4-level CHAID tree with a total of 29 nodes, of which 15 were terminal nodes. Four major predictor variables reached significance to be included in this model including waist circumference, TG, HDL, and FPG. The blood pressure MetS criteria, sex, age, and ethnicity did not reach significance for inclusion in the model. This model had an overall classification accuracy of 92.3% with its ability to detect MetS at 71.8%.

The first level of the tree was split into four initial branches according to the user-specified first level on waist circumference. The mean waist circumference of this sample was 96.82 cm with 49.1% of the total population and 86.6% of the population with MetS presenting with the NCEP waist circumference criteria. The MetS prevalence of subjects whose waist circumference was less than 86 cm was 0.5%, which was significantly less than subjects whose waist circumference was between 86 and 94 cm, between 94 and 103 cm, or greater than 103 cm (8.8%, 21.5%, or 45.8%, resp.).

As seen in the second level of the tree, HDL and TG were shown to be the next best predictor variables for each of the waist circumference splits in the first level. The subset of subjects categorized by a waist circumference less than 86 cm and who had HDL less than or equal to 38 mg/dl had a higher prevalence of MetS (4.8%) than those who had an HDL greater than 38 mg/dl (0.0%). In the subset of subjects with a waist circumference greater than 103 cm, the next split based on HDL of less than or equal to 38 mg/dl and 38–49 mg/dl and greater than 49 mg/dl had MetS prevalence of 82.1%, 45.3%, and 7.9%, respectively.

In the subset of subjects categorized by a waist circumference between 86 and 94 cm the next level based on TG less than 138 mg/dl resulted in lower MetS prevalence (1.6%) compared to TG greater than 138 mg/dl (36.4%). The subset of subjects categorized by a waist circumference between 94 and 103 cm and the next level of TG greater than 162 mg/dl had a MetS prevalence of 57.8% compared to TG less than or equal to 162 mg/dl (5.1% MetS). These results indicate that further testing for MetS might not be warranted for subjects presenting with a waist circumference less than 86 cm but would be recommended for those in either of the subcategories of waist circumference.

FPG level was the most prominent variable in the third level of the tree. The only exception was the split based HDL for subjects who had a waist circumference between 94 and 103 cm and TG level was greater than 162 mg/dl. In the subset of subjects whose waist circumference was between 86 and 94 cm and TG level was less than or equal to 138 mg/dl, FPG less than or equal to 103 mg/dl resulted in 0% MetS prevalence compared to the subset greater than 103 mg/dl (16.7%). This was consistent for subjects who had TG greater than 138 mg/dl with the next level based on FPG less than or equal to 92 mg/dl (0%) compared to FPG greater than 92 mg/dl (52.2%).

In the subset of subjects whose waist circumference was between 94 and 103 cm and TG level was less than or equal to 162 mg/dl, FPG again resulted in a 0% MetS prevalence compared to FPG greater than 162 mg/dl (18.5%). In the subset of subjects whose waist circumference was between 94 and 103 cm and TG level was greater than 162 mg/dl, HDL less than or equal to 38 mg/dl resulted in higher MetS prevalence of 82.6% compared to HDL greater than 38 mg/dl (31.8%). In the subset of subjects whose waist circumference was greater than 103 cm, HDL level greater than 49 mg/dl and FPG greater than 103 mg/dl had a MetS prevalence of 2.1% compared to FPG less than or equal to 103 mg/dl (26.7%). Note that FPG level was the only variable in the fourth level of the tree.

Terminal nodes (nodes that do not split any further) are the ends of each pathway where the prevalence is equated to the likelihood of presenting with MetS. Decision rules for the detection of MetS, presented in Table 2, show the “if-then” logic for each of the 15 terminal nodes. The terminal nodes are chronologically sorted by the proportion of Mets detected, where the highest proportion of 94.4% MetS occurred in node 29 and the lowest proportion of 0% occurred in nodes 6, 14, 16, and 18.

### 3.2. Model Comparison

The following are the results of the user-specified first split model, referred to as the proposed CHAID model, as compared to the CHAID model with no user-specified first split and a logistic regression derived model.

For the CHAID model with no user-specified first split, the first variable was split on FPG. Like the proposed CHAID model, four major predictor variables were selected by the algorithm in this model including waist circumference, TG, HDL, and FPG. The blood pressure MetS criteria, age, sex, and ethnicity did not reach significance and thus were not used in the model. Compared to the proposed CHAID model, this model had a lower, but not practically different, overall classification accuracy of 92.2% with its ability to detect MetS at 69.8%.

The logistic regression model based on the MetS criteria used in CHAID models had no violations of multicollinearity with the model reaching significance. Compared to proposed CHAID model, this logistic regression model had a lower overall classification accuracy of 89.4% with its ability to detect MetS at 61.7%.

## 4. Discussion

The current study aimed to generate a model for the early detection of MetS in young adults. This model was derived using a CHAID algorithm based on the presence of MetS as the target variable and the MetS classification criteria as its predictors whose values were obtained from 2009-2010 NHANES data. MetS is classified by the presence of 3 of 5 criteria defined by either the NCEP or IDF guidelines. The novelty of this study is that the pathways derived from this model show promise in accurately detecting MetS with an easily obtained measurement.

The CHAID model illustrates multilevel interactions among risk factors to identify stepwise pathways to detect MetS. The five variables (waist circumference, TG, HDL, FPG, and blood pressure) were included as predictors of the target variable, MetS. Interestingly, the proposed CHAID model with the user-specified first split on waist circumference outperformed the CHAID algorithm without first-level split specification and the logistic regression model in both overall accuracy and ability to detect MetS.

The user-specified first split on waist circumference in the decision tree was based on the current literature showing that high waist circumference is the most frequent risk component in people with metabolic syndrome [6] and is highly correlated with diabetes and cardiovascular risks [2, 5]. The IDF guidelines use waist circumference as the first criteria followed by two or more other cardiometabolic abnormalities [16]. However, in these guidelines, the waist circumference criteria would be met for MetS if BMI was greater than 30 kg/m^2^ [1]. In the current study, the mean BMI was 29.2 ± 6.8, suggesting that user-specified waist circumference in the decision tree resulted in findings similar to those used by IDF in MetS classification. Two recent studies by Worachartcheewan et al. [20] and Kawada et al. [21] identified the optimal waist circumference cutoff for prediction of MetS. The optimal waist circumference cutoff in the study by Worachartcheewan et al. [20] and Kawada et al. [21] was in the range of 85–88 cm in male and females and 83–85 cm in males, respectively, compared with 86 cm in men and women in the current study. The comparability of these results supports the validity of our findings showing that the CHAID algorithm waist circumference cutoffs could accurately detect MetS.

Central adiposity has been identified as a strong predictor of MetS and a strong contributor to BMI and waist circumference. Després et al. [22] demonstrated a strong correlation between BMI and waist circumference (*r* = 0.91, *P* < 0.05) that is comparable to the current study (*r* = 0.93, data not shown). Furthermore, BMI did not take into consideration the actual body composition, although waist circumference and BMI have been shown to be a strong proxy of visceral adiposity [23]. However, large variances of girth measurements in epidemiological samples weaken the clinical interchangeability between BMI and waist circumference. Waist circumference as compared to BMI might therefore be a more sensitive predictor of MetS, especially in the at-risk young adult population. A waist circumference screening could more readily and easily alert health providers to the increased metabolic risks associated with excessive visceral fat accumulation over other MetS classification criteria that require fasting, blood draws, and analysis. Therefore waist circumference shows promise as an initial predictor in the detection of MetS prior to further testing.

Interestingly, blood pressure did not reach significance to be included in the final model. One possible explanation is that elevations in blood pressure are less prevalent in younger adults [13]. Within our sample, the blood pressure criteria had the lowest prevalence of all the MetS classification criteria for subjects with and without MetS (10.1% and 0.8%, resp.).

### 4.1. Limitations

The current study was intended as a pilot study meant to explore and test the CHAID algorithm's utility in creating pathways to detect MetS in young adults. Although this model had an overall accuracy of 92.3%, its ability to accurately detect MetS was only 71.8%. The CHAID algorithm requires large sample sizes to operate effectively. Given that the parent and child nodes were set to split at small sizes (20 and 5, resp.) and that there was no validation of the model, the derived pathways for MetS detection from this study are not intended for clinical use. Furthermore, the MetS diagnosis in this analysis was not a clinical diagnosis but was rather determined by the presence of three or more of the NCEP criteria based on their prevalence within the secondary data set. Additionally, this analysis did not account for the use of medications to control blood pressure, lipids, and/or plasma glucose.

The CHAID analysis did not identify any significant differences in MetS based on sex or ethnicity in this sample although previous studies have shown differences in MetS risk based on sex and ethnicity [24, 25]. Considering the limitation of the current study, future investigations warrant utilizing sufficiently large sample sizes, considering the difference in MetS based on the sex and ethnicity and performing model validation.

## 5. Conclusion

In summary, these preliminary findings suggest that young adults at risk for MetS, who are not routinely screened for fasting blood lipids or FPG, could be identified for further follow-up testing based on their waist circumference. Future research warrants the investigation of other anthropometric measures, simple point-of-care techniques, and validation of these decision tree methods to create a strong algorithm for predicting and/or the early detection of MetS in young adults. There are no clinically established criteria for premetabolic syndrome. Decision tree methods are promising regarding preliminary MetS detection and can aid in the development of a formal definition of premetabolic syndrome. If established, premetabolic syndrome diagnostic criteria could improve outcomes associated with the development of MetS or could halt the progression of MetS and its relative consequences.

## Figures and Tables

**Table 1:** Subject demographics and descriptive statistics.

Parameter	Mean ± standard deviation (*n* = 745)
Age (yr)	29.3 ± 5.8
Weight (kg)	82.7 ± 21.3
Height (cm)	168.2 ± 9.9
Body mass index (kg/m^2^)	29.2 ± 6.8
Systolic blood pressure (mmHg)	113.8 ± 11.7
Diastolic blood pressure (mmHg)	66.7 ± 11.8
Waist circumference (cm)	96.8 ± 15.8
HDL (mg/dl)	51.30 ± 14.9
Triglyceride (mg/dl)	126.7 ± 114.3
Fasting plasma glucose (mg/dl)	98.0 ± 24.6

Values are mean ± standard deviation. HDL: high-density lipoprotein cholesterol (*n* = 745; male = 335, female = 410).

**Table 2:** Decision rules for the prediction of the incidence risk of MetS from the CHAID algorithm.

Node number	Level 1	Level 2	Level 3	Level 4	MetS probability
29	94 < waist circumference ≤ 103	TG > 162	HDL ≤ 38	FPG > 94	94.4
11	Waist circumference > 103	HDL ≤ 38	∗	∗	82.1
17	86 < waist circumference ≤ 94	TG > 138	FPG > 92	∗	52.2
12	Waist circumference > 103	38 < HDL ≤ 49	∗	∗	45.3
28	94 < waist circumference ≤ 103	TG > 162	HDL ≤ 38	FPG ≤ 94	40.0
21	94 < waist circumference ≤ 103	TG > 162	HDL > 38	∗	31.8
27	Waist circumference > 103	HDL > 49	FPG > 103	∗	26.7
19	94 < waist circumference ≤ 103	TG ≤ 162	FPG > 99	∗	18.5
15	86 < waist circumference ≤ 94	TG ≤ 138	FPG > 103	∗	16.7
5	Waist circumference ≤ 86	HDL ≤ 38	∗	∗	4.9
26	Waist circumference > 103	HDL > 49	FPG ≤ 103	∗	2.1
6	Waist circumference ≤ 86	HDL > 38	∗	∗	0.0
14	86 < waist circumference ≤ 94	TG ≤ 138	FPG ≤ 103	∗	0.0
16	86 < waist circumference ≤ 94	TG > 138	FPG ≤ 92	∗	0.0
18	94 < waist circumference ≤ 103	TG ≤ 162	FPG ≤ 99	∗	0.0

*represents not significant. Growing method: exhaustive CHAID; dependent variable: MetS: metabolic syndrome, TG: triglyceride, HDL: high-density lipoprotein cholesterol, and FPG: fasting plasma glucose.
